# Every School Healthy: Policy, Research, and Action

**DOI:** 10.1111/josh.12954

**Published:** 2020-12

**Authors:** Nora L. Howley, Holly Hunt

**Affiliations:** a Lerdau, LLC, 8710 Cameron Street, #817, Silver Spring, Maryland 20910.; b US Centers for Disease Control and Prevention, Division of Population Health, School Health Branch, Atlanta, Georgia.

The invitation to serve as guest editors of a sponsored issue, such as this one is a privilege, and it comes with a responsibility to showcase the impactful work of diverse and talented authors and colleagues. This responsibility also includes the need to ensure the content reflects the values of the American School Health Association, the Robert Wood Johnson Foundation, and its Together for Healthy and Successful Schools program grantees (with whom the idea for this issue originated).

As we write this in the summer of 2020, we acknowledge that the world has changed a lot from the end of 2019 when this issue was first conceptualized and early 2020 when the authors were invited and articles were underway. We also know that it will have changed even more by the time this is published. Some of the biggest changes have been and will continue to be in the complicated and interlocking systems of education and health.

We believe that these articles continue to be relevant, but it is important to acknowledge that they reflect work performed in very different conditions. We think it is fair to say that neither we nor any of the contributors to this issue could imagine the changes being wrought in the nation’s schools and surrounding communities by COVID-19. At the same time, the powerful response to our nation’s history of racism and inequality, much of it being led by young people, is leading to even more changes—particularly in areas of interest to readers of this special issue, such as the social-emotional and physical environments of schools.

As you read these articles and consider their implications for your work in supporting healthy and safe environments for young people, we ask that you also reflect on the larger implications. What do they have to say for our future and the future of our young people? What are our responsibilities for creating systemic and systematic changes that will make schools healthier and safer for all?

## Creating this Issue of the Journal of School Health

Our starting place for this issue was the last sponsored issue of the *Journal of School Health*, published in November 2015. That issue, which one of us (Hunt) co-edited, introduced the Whole School, Whole Community, Whole Child (WSCC) model to the field. The WSCC model was “… created to encourage education and health organizations to work together to improve student health and academic outcomes”^1^ based on the strong bi-directional relationship between the two sectors. By encompassing the individual student, the family, the school, and the community-at-large, the WSCC model offers opportunities to impact the conditions in schools that influence healthy behavior and learning.^2^

The WSCC model can be a useful tool for the building of a Culture of Health. As conceptualized by the Robert Wood Johnson Foundation (RWJF), a Culture of Health is one in which everyone has a fair and just opportunity for health and well-being. In its *Together for Healthy and Successful Schools* program, the RWJF set out to explore how, with equity as the undergirding principle, the WSCC model could be used to build a Culture of Health in schools. Three areas of interconnected work, Research, Policy, and Strategic Action and Alignment were identified as key to doing so.^3^ The accompanying guest editorial in this issue by Elstein and Ng’andu offers a deeper look at the RWJF’s perspective.^4^

With 3 lead grantees (each with partners) designated to work in these areas above, this effort sought to catalyze new work, some of it by groups or individuals who had been actively engaged in supporting healthy environments for students, but in areas outside of the traditional school health field. The resulting products and lessons learned from this initiative are now being disseminated and utilized throughout the nation. They are available to all at healthysuccessfulschools.com.

The work of these grantees and their partners serves as the foundation for this issue. We began with six articles that emerged from the innovative work of these grantees—America’s Promise Alliance, Child Trends, and Health Equity Works. We then sought to obtain eight additional articles that would complement and expand on the work of the RWJF initiative. We use the word “sought” because these articles were selected through a process that cast a wide net and offered potential authors the opportunity to submit an abstract before undertaking the writing of a full article. In this process we sought:

- Articles that centered questions of equity in both education and health within a WSCC approach—we believe that it is only when we, as a field, explicitly addresses equity that it can drive the needed changes.
- Articles that described the interconnectedness and coordination of more than one aspect or component of the WSCC model—we believe that to drive change, all elements of the model should be incorporated.
- Articles by individuals whose work may not be well known to the field of school health or who may not have previously published in the *Journal*—we believe that by exploring novel efforts not previously shared and in related fields we may find opportunities otherwise missed to elevate and support positive changes.

We received over 40 submissions, and whereas selection was challenging, we believe that this selection of articles provides a comprehensive picture of WSCC in action. It is by no means inclusive of all the many novel and promising approaches, but represents a small number of those that have been impactful. Taken together, they offer both questions and answers to inform the field.

Before moving on to the articles, we want to note the relevance of the open access role of this issue of the *Journal of School Health*. Much of the power and influence of the 2015 sponsored issue centered on the WSCC approach can be attributed to its availability through open access for all (supported by the US Centers for Disease Control and Prevention). The RWJF has made the same commitment to provide open access for this issue. It is our hope that the contents will positively influence and contribute to healthy environments for students and adults in schools, as the previous sponsored issue did.

## What Do We Mean by Equity?

We know that the term equity can mean different things to different people. In the context of this work, equity is about opportunities. A 2017 RWJF report stated: “[H]ealth equity means that everyone has a fair and just opportunity to be as healthy as possible. This requires removing obstacles to health such as poverty, discrimination, and their consequences, including powerlessness and lack of access to good jobs with fair pay, quality education and housing, safe environments, and health care.”^2^ Education equity can be thought of in the same way. The National School Boards Association states that equity is “when all students receive the resources they need so they graduate prepared for success after high school.”^5^

## Connections

Intentional consideration of the resources can support the offering of fair and just opportunities. These resources are not just financial. They are the policies and programs, the strategies, and the people who are brought together to support the health and well-being of students through the WSCC approach. These articles, all of which seek to address equity, do not offer easy solutions. Rather, they offer a wide range of perspectives, ideas, and observations for the field’s consideration. And, whereas the article could stand alone, we see them as deeply connected. We hope that as you read these, you will see your own connections between the articles and your work.

At the heart of all of the articles is the role of systems. WSCC is neither a cookie-cutter model nor a set of boxes to be checked. Rather, it is a set of connected components, people, and places centered on the student. We do not think it unreasonable to note that almost any school or district has most, if not all, 10 components in place. A WSCC approach requires that we look at both how the components are coordinated at all levels and the role of the community in that coordination.

Throughout the issue, common themes arise, along with some unique initiatives that show promise and give us pause to think about new and different approaches. As one example, we can look at the work of McMullen et al.,^6^ who, using the research strategy of a systematic review, note that there has been little empirical research on effective strategies for community engagement, a core component of WSCC. Similarly, the articles by Purnell et al.^7^ and Ballard et al.^8^ apply tools new to this field, such as Network Analysis and Group Model Building, to understanding the systems within school communities. Their findings lay the groundwork for further work to better understand how to build and sustain truly authentic community engagement.

The role of youth engagement and youth perspectives in a WSCC model is another area of examination. Sprague Martinez et al.^9^ explore how schools and community partners can create the opportunities for authentic, ongoing work by young people—not only to participate, but also to lead. Moreover, as discussed below, Bottiani and Henderson^10^ explore how young people in one urban area relate to and influence adults within their schools.

Having good policies in place is a necessary, but not sufficient condition for sustainable changes in schools. The WSCC model identifies coordination as essential to policy implementation. Policy plays a major role in several of the articles in this issue. Chiriquí et al.^11^ report on the results of a study of local policies in 20 states across all 10 components of WSCC. By comparing the policies of local school districts with their respective state’s policies, they are able to identify areas of policy gaps and/or lags—concluding that policy change in support of WSCC may need to be bi-directional, with both states and districts as sites of policy advocacy.

The works of Mays^12^ and Koriakan et al.^13^ both look at federal policies that can improve WSCC implementation. The former describes how some states are leveraging Medicaid to improve access in several WSCC areas including health services and counseling, psychological and social services.^12^ The latter discusses the development of a tool, WellSTAT WSCC, that grew out of the federal-local wellness policy mandate.^13^ By moving beyond the requirements of the mandate and embracing all the components of WSCC, this tool offers school districts a way to think more comprehensively about their local wellness policy, a strategy addressed by Baldwin and Ventresca.^14^

But policy in and of itself is not enough to create healthy conditions. As Temkin et al.^15^ point out, the issue of student experience of trauma is not amenable to quick policy fixes. Indeed, ill-conceived policy may exacerbate student experiences with trauma. They argue for policymakers to take a holistic approach that recognizes the limits of the evidence base in this area.

The adults who engage with young people matter, as does how young people view them. Pittman et al.^16^ examine some of the “unusual suspects,” in particular those outside the time and space of the instructional classroom, such as recess, before/after school, and summer programs. Supported by the emerging knowledge of brain development, they make the argument that these adults are important to true WSCC implementation.^16^

Bottiani and Henderson^10^ illustrate the importance and influence of young people’s perspectives on adults. The role of supportive relationships with key adults in the school building, particularly those relationships that offer cultural responsiveness and caring, is highlighted. Their findings about how these relationships can buffer students of color from the impacts of structural racism offer important considerations for policymakers and school leaders.^10^

In related work, Gaias et al.^17^ report on the findings of an intervention specifically designed to address teacher implicit biases and improve the school climate for all students, particularly students of color. Again, their findings about the need to build the capacity of teachers through training and professional development offer guidance for policymakers and administrators.

The final set of articles provide examples of putting WSCC implementation into practice. At the national level, Pufall Jones et al.^18^ offer important insights into the role of organizations, particularly national ones, in offering technical assistance to school districts and communities. Their findings on the need for coordination and flexibility are valuable insights into the future of taking WSCC to scale.

Baldwin and Ventresca^14^ describe the complexity of WSCC in a large urban school district. Their case-study of district-wide implementation highlights how the district uses data to engage stakeholders and to identify gaps in programs. In this case, their attention is on students with special education needs and those identified as English Language Learners and the need, through partnerships, to tailor and provide comprehensive sexual health education that is accessible to these students.^14^

Shattuck et al.^19^ focus on another group of often overlooked students, sexual, and gender minority youth. In this case, they focus on how the adults working to support these students understand the factors that impact implementation of supportive policies and practices. Through listening to these adults, the authors highlight the need for cultural competence not just at the personal level, but at the structural (school and district), too, in order to advance supportive policies into sustainable practices.^19^

## What Else, What Next?

We recognize that no single issue of a journal can address all of the many innovative approaches taken and valuable lessons learned - not to mention the enormously varied roles of key stakeholders. In this final section, we suggest areas that, based on the articles in this issue and the abstracts we received in the early stages, can use further attention and exploration to advance equity and impact healthy outcomes for students. We propose continued attention to:

- the needs of students with special educational needs;
- the needs of non-English speaking students and their families;
- the stakeholders within the school, including support staff members, those working for outside agencies (such a partner non-profits), and volunteers;
- the systems that operate in schools, districts, and communities to identify points of leverage and challenge;
- the factors that make a school physically and emotionally safe for young people, particularly young people of color;
- the youth voices and perspectives across all components; and
- the conditions in communities that create opportunities and barriers to equity.

In addition, because a WSCC approach is not a cookie-cutter model, we think it worthwhile to continue to explore how tools such as the *Healthy Schools Toolkit* and WellSat WSCC—supported by technical assistance at all levels—can drive change.

## Conclusion

As evidenced by the experience and learnings of the authors, the WSCC approach has led to success in creating a better understanding of how the components and key stakeholders within and around a school play a role in creating healthy environments for students. When equity is centered in WSCC, great things can and will happen for young people and those who work with and care for them in schools and communities.

We close with a set of thanks to those who made this issue possible. First of all, we thank the authors for their hard work and their insightful findings. Second, we thank the American School Health Association and the *Journal of School Health* for giving all of us this platform. Third, we thank our numerous colleagues who provided feedback and support through this process. And, finally, we express our gratitude to the Robert Wood Johnson Foundation for inviting us to take on this project and for its unwavering commitment to building a true Culture of Health in our country.
